# Intermittent Hypoxia Severity in Animal Models of Sleep Apnea

**DOI:** 10.3389/fphys.2018.01556

**Published:** 2018-11-06

**Authors:** Ramon Farré, Josep M. Montserrat, David Gozal, Isaac Almendros, Daniel Navajas

**Affiliations:** ^1^Unitat de Biofísica i Bioenginyeria, Facultat de Medicina i Ciències de la Salut, Universitat de Barcelona, Barcelona, Spain; ^2^CIBER de Enfermedades Respiratorias, Madrid, Spain; ^3^Institut Investigacions Biomèdiques August Pi Sunyer, Barcelona, Spain; ^4^Sleep Lab, Hospital Clínic de Barcelona, Barcelona, Spain; ^5^Department of Child Health, University of Missouri School of Medicine, Columbia, MO, United States; ^6^Institute for Bioengineering of Catalonia, The Barcelona Institute of Science and Technology, Barcelona, Spain

**Keywords:** intermittent hypoxia, oxygen dissociation curve, hypoxia/reoxygenation, sleep breathing disorders, disease model animal, obstructive sleep apnea syndrome

Obstructive sleep apnea (OSA) is a very prevalent breathing disorder (Peppard et al., 2013; Heinzer et al., 2015) characterized by recurrent obstructions of the upper airway during sleep. These obstructions, which can present as either frank apneas or hypopneas, result in cyclic events of arterial hypoxemia with or without hypercapnia since blood circulating through lung capillaries during the obstructive events does not receive the required amount of oxygen because interrupted ventilation reduces the partial pressure of oxygen (PO_2_) in alveolar air. Intermittent arterial hypoxemia immediately translates into the systemic capillary circulation with the result that all patient's tissues are subjected to cycles of hypoxia/reoxygenation of varying severity (Almendros et al., 2010, 2011, 2013; Reinke et al., 2011; Torres et al., 2014; Moreno-Indias et al., 2015). There is now ample experimental and epidemiological evidence that the oxidative stress and inflammatory cascades elicited at both systemic and tissue levels by the recurrent hypoxia-reoxygenation events are major drivers of the clinically relevant morbid consequences of OSA (Lavie, 2015), namely increased risk of cardiovascular, metabolic, neurocognitive and malignant diseases (Lévy et al., 2015). Given the major health care burden posed by OSA and the ethical impossibility of carrying out precise mechanistic studies focused on causality in patients with OSA, considerable efforts have been preferentially focused on animal models of OSA (Davis and O'Donnell, 2013; Chopra et al., 2016) and more particularly on those mimicking intermittent hypoxemia.

To realistically model intermittent hypoxemia in OSA it is important to bear in mind that the potential effect of this exposure depends on the magnitude of the decrease in arterial oxygen partial pressure (PaO_2_), since this biological variable *de facto* determines the PO_2_ gradient across the capillary membrane, and hence oxygen delivery to cells within the various tissues. In practice, the conventional setting for subjecting animals to intermittent hypoxia consists of cyclically changing the oxygen fraction (FiO_2_) in the environmental gas breathed by the animals from room air (F_I_O_2_ - 21%) to different nadir values ranging from F_I_O_2_ of 4 to 15%, and thus model different degrees of hypoxia severity which, as would be anticipated, yield dose-response effects (Nagai et al., 2014; Lim et al., 2016; Gallego-Martin et al., 2017; Docio et al., 2018). When using this experimental setting the degree of intermittent hypoxemia achieved for a given FiO_2_ swing is similar in young and old animals (Dalmases et al., 2014) which is interesting given that the effects of hypoxia/reoxygenation are modulated by age (Torres et al., 2018). The conventional settings used for applying intermittent hypoxia to animals include two major parameters, with one of them being the frequency of hypoxic events [which loosely corresponds to the clinical index of apnea-hypopnea index (AHI) recorded in polysomnographic studies of patients with OSA, Berry et al., 2018]. Setting the frequency of intermittent hypoxia to mimic different values of AHI is a relatively straightforward proposition, and different investigators have carried out animal experiments in which they modeled varying rates of hypoxic events such as to cover a wide range of AHI, e.g., from the highest events rates in OSA patients (60 events/h) to occasional events (2 events/h) (Almendros et al., 2013; Shiota et al., 2013; Dalmases et al., 2014; Jun et al., 2014; Briançon-Marjollet et al., 2016; Gozal et al., 2017). Moreover, at any given frequency of cycling it is also simple to prescribe different durations for the de-oxygenation and re-oxygenation phases within each cycle (Chodzynski et al., 2013; Lim et al., 2015).

The second major index defining the characteristics of the experimental OSA paradigm is the severity of the hypoxic stimulus within each event. Determining the severity of the actual intermittent hypoxemia in OSA patients by measuring the variable which is physiologically relevant in terms of oxygen transport to tissues in systemic capillaries (PaO_2_) is not easy, particularly when taking into account that in OSA this variable changes at a high frequency. Such real time measurements would only be possible if using fast fiberoptic oxygen sensors suitable for human use and capable of continually measure fast variations of oxygen partial pressure in blood (Formenti et al., 2015) or by a technique of repetitive blood sampling along the obstructive events as carried out in animals (Lee et al., 2009). However, such invasive or complex measurements are clearly not appropriate for OSA patients in clinical settings, especially when considering that indirect estimates of PaO_2_ can be easily and non-invasively obtained by means of pulse oximetry. Indeed, this technique –which provides real time measurement of SaO_2_ (i.e., the percentage of oxy-hemoglobin in arterial blood within each heartbeat) – is an accurate surrogate of PaO_2_ since for conventional conditions (e.g., pH, temperature) the oxygen dissociation curve of human blood provides a direct relationship between PaO_2_ and SaO_2_ (Severinghaus, 1979). Given the ease of its measurement, pulse oximetry is currently the standard tool to clinically quantify the severity and duration of hypoxic events in OSA patients (Berry et al., 2018). Fortunately, pulse oximetry can be also applied to measure SaO_2_ in animals. Indeed, given that hemoglobin from different species presents virtually the same absorption properties in the visible and near infrared regions (Grosenbaugh et al., 1997), pulse oximeters for small animals only require software adaptations to deal with much higher heart rate frequencies than in humans, even if they make inferential assumptions and annul considerations regarding potential differences across saturation equations in rodents. Nevertheless, characterizing the depth of the hypoxemic events in animal models of OSA and compare them with those in patients is not straightforward, and constitutes a controversial issue in sleep apnea research. Furthermore, the potential presence of hemodynamic instability, tissue perfusion changes, and accuracy issues of oximeters at the low ranges of saturation need to be considered as well. This Opinion article aims to contribute to this topic discussion by illustrating important considerations and evidence in this contextual setting.

The major controversial point refers to one of the most commonly employed paradigms of intermittent hypoxia when applied to mice, and resulting in measured SaO_2_ swings with nadir values in the range 50–70% (Jun et al., 2010; Reinke et al., 2011; Torres et al., 2014, 2015; Lim et al., 2015, 2016). Specifically, in a recent review focused on sleep apnea research in animals, it was stated that such intermittent hypoxia profiles may result in significantly more severe hypoxic events than those typically experienced by patients with OSA, in whom SaO_2_ nadir ranges of 77–90% are usually documented (Lim et al., 2015), and that therefore extrapolation from murine models to human disease should be applied with caution (Chopra et al., 2016). As will become apparent from the following discussion, we disagree with such an objection, and instead state that such low SaO_2_ nadirs (50–70%) in mice realistically mimic the hypoxemic events in patients since they actually correspond to PaO_2_ values similar to those in OSA patients. The rationale behind such assertion is that SaO_2_ is misleading when comparing severity of hypoxemia across different species because the oxygen dissociation curve in mice is considerably different from humans, and this point will be reinforced while we elaborate on well-established physiological data depicting oxygen dissociation curves across species.

The top of Figure 1 shows a representation of the typical SaO_2_-PaO_2_ relationships in human (red line) and mouse (blue line). The usually overlooked fundamental fact that there is a shift of the oxygen dissociation curve toward the right as the body size of the species decreases was already firmly established 60 years ago (Schmidt-Neilsen and Larimer, 1958). More specifically, half-saturation pressure (P_50_), defined as PaO_2_ corresponding to SaO_2_ = 50%, and typical body weight (BW) of any mammalian species were shown to be closely prescribed by a classical heterogonic relationship (Adolph, 1949) in biological scaling (Schmidt-Nielsen, 1970): P_50_ = 50.34 × BW^−0.054^ (P_50_ in mmHg, BW in grams), for a range of mammals encompassing horse to mouse (Schmidt-Neilsen and Larimer, 1958). It is also remarkable that the Bohr effect (shifting of the dissociation curve as blood CO_2_/pH increases/decreases) applies to mammalian blood with a magnitude range that also closely follows a heterogonic relationship (Riggs, 1960), a factor that could further amplify the human-mouse difference, particularly when CO_2_ increases during obstructive apneas are taken into account (Farré et al., 2007). Interestingly, the earlier reports on detectable differences among hemoglobin dissociation curves in human and mouse blood have been subsequently confirmed as measurement techniques have improved (Gray and Steadman, 1964; Severinghaus, 1979; Mouneimne et al., 1990; Geng et al., 2016). Figure 1 (top) clearly shows that for any given SaO_2_ value, PaO_2_ is always lower for human than for mouse blood. In other words, mice have more oxygen reserve than humans. These data can be plotted as the relationship between SaO_2_ in mice and humans for any value of PaO_2_ as shown in the bottom panel of Figure 1, illustrating that SaO_2_ in mice is always below the identity line. This figure shows that the typical range of SaO_2_ nadir values in patients (70–90%) corresponds to nadir SaO_2_ values in the range 45–72% in mice, thereby firmly confirming the adequacy of current experimental environmental hypoxia protocols used in murine models.

**Figure 1:**
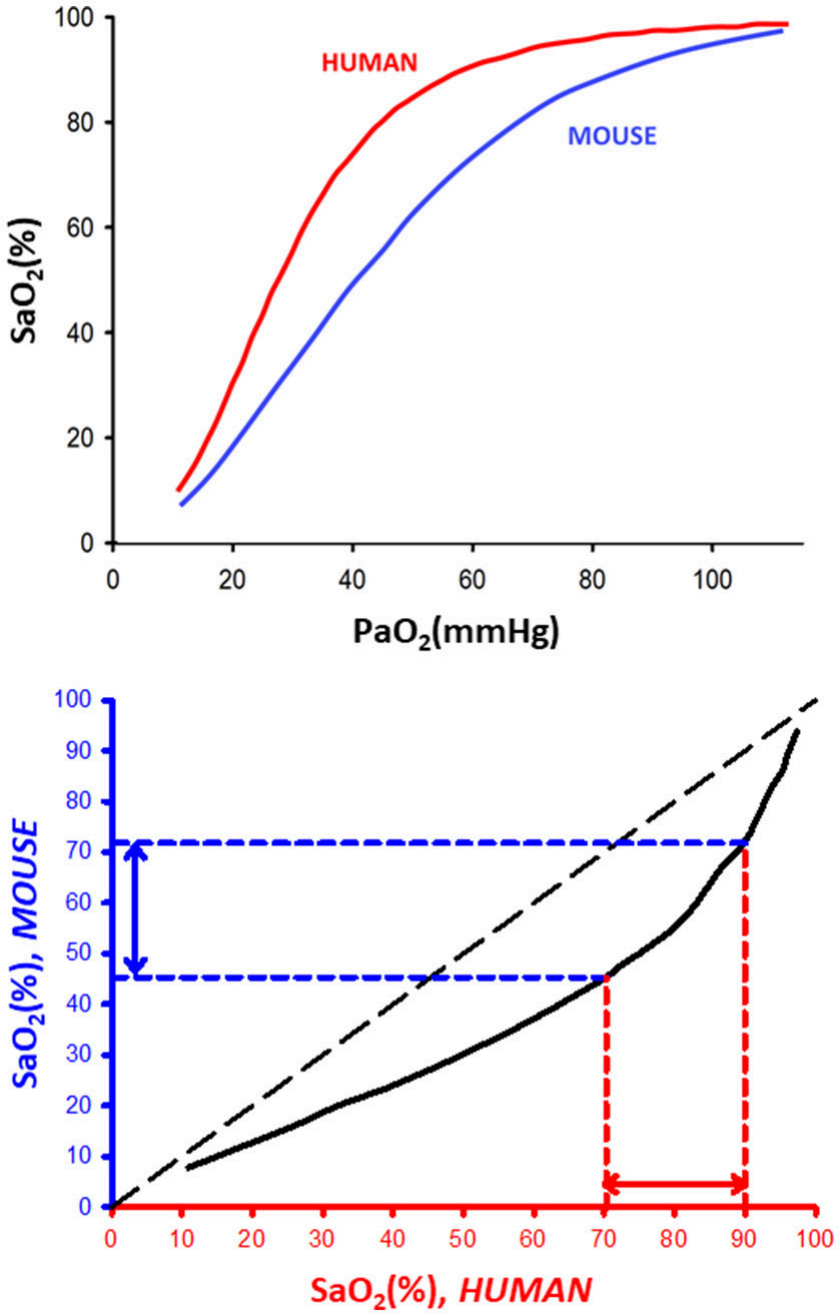
**(Top)** Representative oxygen dissociation curves for human and mouse blood. SaO_2_ and PaO_2_: arterial oxygen saturation and partial pressure, respectively. The dissociation curves for human and mouse blood were redrawn from data in Severinghaus (1979) and Geng et al. (2016), respectively. **(Bottom)** Relationship between equivalent (same PaO_2_) values of SaO_2_ in human and mouse blood.

In conclusion, to achieve PaO_2_ nadir values similar to those traditionally experienced by severe OSA patients –i.e., to realistically simulate patients' hypoxia– SaO_2_ in mice should be much lower than the SaO_2_ observed in patients. These considerations substantiate our initial objection to the statement by Chopra and coauthors (Chopra et al., 2016) that the currently used experimental paradigms of intermittent hypoxia (SaO_2_ swings with nadir 50–70%) in mice are not at all excessively severe, but rather closely match human conditions. Therefore, current intermittent hypoxia exposure paradigms adequately and reliably perform when subjecting mouse cells in the various tissues to a realistic hypoxia/reoxygenation challenge that enables investigation of mechanisms underlying the end-organ adverse consequences of OSA.

## Author contributions

RF conceived and drafted the manuscript. JM, DG, IA, and DN provided their expertise in animal models, respiratory physiology and pathophysiology of sleep apnea, and significantly contributed to write the final version.

### Conflict of interest statement

The authors declare that the research was conducted in the absence of any commercial or financial relationships that could be construed as a potential conflict of interest.
